# Impact of penicillin allergy labels on surgical site infections in a large UK cohort of gastrointestinal surgery patients

**DOI:** 10.1093/jacamr/dlae022

**Published:** 2024-02-16

**Authors:** Nick K Jones, Brian Tom, Constantinos Simillis, John Bennet, Stavros Gourgiotis, Jo Griffin, Helen Blaza, Shuaib Nasser, Stephen Baker, Theodore Gouliouris

**Affiliations:** Department of Medicine, University of Cambridge, Cambridge, UK; Cambridge Clinical Microbiology and Public Health Laboratory, Cambridge University Hospitals NHS Foundation Trust, Addenbrooke’s Hospital, Hills Road, Cambridge CB2 0QQ, UK; Department of Infectious Diseases, Cambridge University Hospitals NHS Foundation Trust, Cambridge, UK; MRC Biostatistics Unit, Cambridge University, Cambridge, UK; Cambridge Colorectal Unit, Cambridge University Hospitals NHS Foundation Trust, Cambridge, UK; Department of Surgery, University of Cambridge, Cambridge, UK; Department of General Surgery, Cambridge University Hospitals NHS Foundation Trust, Cambridge, UK; Department of General Surgery, Cambridge University Hospitals NHS Foundation Trust, Cambridge, UK; Department of Infection, Prevention and Control, Cambridge University Hospitals NHS Foundation Trust, Cambridge, UK; Department of Infection, Prevention and Control, Cambridge University Hospitals NHS Foundation Trust, Cambridge, UK; Department of Allergy, Cambridge University Hospitals NHS Foundation Trust, Cambridge, UK; Department of Medicine, University of Cambridge, Cambridge, UK; Department of Medicine, University of Cambridge, Cambridge, UK; Cambridge Clinical Microbiology and Public Health Laboratory, Cambridge University Hospitals NHS Foundation Trust, Addenbrooke’s Hospital, Hills Road, Cambridge CB2 0QQ, UK; Department of Infectious Diseases, Cambridge University Hospitals NHS Foundation Trust, Cambridge, UK

## Abstract

**Objectives:**

Studies in the USA, Canada and France have reported higher surgical site infection (SSI) risk in patients with a penicillin allergy label (PAL). Here, we investigate the association between PALs and SSI in the UK, a country with distinct epidemiology of infecting pathogens and range of antimicrobial regimens in routine use.

**Methods:**

Electronic health records and national SSI surveillance data were collated for a retrospective cohort of gastrointestinal surgery patients at Cambridge University Hospitals NHS Foundation Trust from 1 January 2015 to 31 December 2021. Univariable and multivariable logistic regression were used to examine the effects of PALs and the use of non-β-lactam-based prophylaxis on likelihood of SSI, 30 day post-operative mortality, 7 day post-operative acute kidney injury and 60 day post-operative infection/colonization with antimicrobial-resistant bacteria or *Clostridioides difficile*.

**Results:**

Our data comprised 3644 patients and 4085 operations; 461 were undertaken in the presence of PALs (11.3%). SSI was detected after 435/4085 (10.7%) operations. Neither the presence of PALs, nor the use of non-β-lactam-based prophylaxis were found to be associated with SSI: adjusted OR (aOR) 0.90 (95% CI 0.65–1.25) and 1.20 (0.88–1.62), respectively. PALs were independently associated with increased odds of newly identified MRSA infection/colonization in the 60 days after surgery: aOR 2.71 (95% CI 1.13–6.49). Negative association was observed for newly identified infection/colonization with third-generation cephalosporin-resistant Gram-negative bacteria: aOR 0.38 (95% CI 0.16–0.89).

**Conclusions:**

No evidence was found for an association between PALs and the likelihood of SSI in this large UK cohort, suggesting significant international variation in the impact of PALs on surgical patients.

## Introduction

Surgical-site infections (SSIs) are the third commonest cause of healthcare-associated infection.^1^ Gastrointestinal surgery is particularly high risk, with SSI incidence in England estimated to be 1.9% after gastric surgery, 7.4% after small bowel surgery, 8.6% after colorectal surgery and 15.4% after hepato-biliary (HPB) surgery.^2^ Antimicrobial prophylaxis is used as an effective means of preventing SSI in patients undergoing selected procedures, accounting for approximately 14% of hospital antimicrobial consumption in Europe^3^ and 8% in England.^4^ Despite a wealth of randomized controlled trial data to support the use of antimicrobials in colorectal surgery,^5^ evidence relating to other surgery types is weaker, and no particular regimens have been found to have superior efficacy for any procedure type,^5–13^ with very rare exceptions.^6^ As a result, national and international consensus on the optimal use of antimicrobial prophylaxis is lacking, and practice varies significantly between institutions.

A major factor in determining the choice of prophylactic regimen is the presence of recorded antimicrobial allergy. It is estimated that 5%–15% of individuals in developed countries are labelled as penicillin allergic, making penicillin the most commonly reported antibiotic allergen.^14,15^ Penicillin allergy labels (PALs) have been associated with a wide range of adverse health outcomes,^16–29^ but the vast majority of labels are thought to be spurious, and penicillin allergy ‘de-labelling’ programmes are emerging as promising antimicrobial stewardship tools.^30–32^ Recent single-centre retrospective cohort studies in the USA, Canada and France have reported increased odds of SSI in patients with a PAL, which is thought to be mediated by the use of inferior, non-β-lactam-based prophylaxis.^33–41^ However, it is unknown whether these findings are generalizable to the UK, where the epidemiology of infecting pathogens, prevalence of antimicrobial resistance (AMR) and range of routinely used prophylactic regimens differ. Here, we investigate whether there is association between PALs, or non-β-lactam-based prophylaxis, and SSI in a large hospital cohort of gastrointestinal surgery patients in England.

## Patients and methods

The retrospective cohort comprised all patients undergoing gastrointestinal surgery at Cambridge University Hospitals NHS Foundation Trust (CUH) and enrolled in a national programme of SSI surveillance between 1 January 2015 and 31 December 2021.^42^ CUH is a 1100-bed university teaching hospital that provides elective and emergency surgical services, including for solid organ transplantation. All hospital admissions are routinely screened for MRSA colonization via culture from multisite swabs (nose, throat and groin/perineum). Routine screening for carbapenemase-producing Enterobacteriaceae (CPE) or VRE colonization via culture from rectal swabs or faecal samples is reserved for a small number of high-risk patient groups, including prospective or past organ transplant recipients, individuals with prior hospitalization in locations with high CPE prevalence, and those admitted to critical care areas. Routine ESBL screening is reserved for neonates on the neonatal ICU. SSI surveillance data had been collected prospectively, following a nationally standardized protocol,^42^ by designated nurses trained by the UK Health Security Agency (UKHSA, formerly PHE) national coordinating centre. Patient names, dates of birth, NHS numbers and dates of surgery were used as unique identifiers to match data extracted from the UKHSA SSI surveillance service (SSISS) database and the hospital’s electronic health records system (Epic) on 28 March 2022.^43^

### Exposure

The presence of a PAL at the time of surgery was ascertained from the ‘allergy’ section of Epic, having filtered for entries relating to the penicillin antimicrobial class and specific named penicillin-containing agents. Adverse reactions of any type or severity were included in the exposure definition, including those coded as ‘intolerance’ or ‘contraindication’. In sensitivity analyses, the exposure definition was restricted to labels with the reaction type recorded as ‘allergy’, ‘unknown/unable to establish’, or left blank, and the use of non-β-lactam-based prophylaxis was investigated as a separate exposure. CUH implements prescriptive antimicrobial prophylaxis guidelines for patients with and without PALs, which vary according to procedure type (Table S1, available as Supplementary data at *JAC-AMR* Online). These guidelines did not change during the observation period of this study.

### Outcomes

The primary outcome was inpatient SSI within 30 days of surgery, which was obtained from the UKHSA SSISS database. This had been recorded via active inpatient surveillance until 30 days post-surgery or the date of hospital discharge, whichever came sooner.

Secondary outcomes were post-operative mortality within 30 days, post-operative acute kidney injury (AKI) within 7 days, newly identified infection/colonization with MRSA within 60 days after surgery, newly identified infection/colonization with VRE within 60 days after surgery, newly identified infection or colonization with third-generation cephalosporin-resistant Gram-negative bacteria within 60 days after surgery, and newly identified infection/colonization with *Clostridioides difficile* within 60 days after surgery.

Inpatient and outpatient mortality data were extracted from Epic. AKI data were taken from the ‘AKI alert’ function in Epic, which defines AKI as any rise in serum creatinine ≥26 μmol/L within a 48 h period or a ≥50% rise from baseline. The ‘AKI alert’ function came into use in January 2017, so procedures from 2015 and 2016 were excluded from analyses involving AKI.

Microbiology data were extracted from Epic and its Laboratory Information System, Epic-Beaker. Positive MRSA status was determined by the presence of either a *Staphylococcus aureus* culture result with ‘resistant’ as the flucloxacillin susceptibility result, or an ‘MRSA’ infection control flag. Positive VRE status was determined by the presence of either an *Enterococcus* species culture result with ‘resistant’ for the vancomycin susceptibility, or a ‘VRE’ infection control flag. Positive third-generation cephalosporin-resistant Gram-negative status was determined by the presence of either an Enterobacterales culture result with ‘resistant’ for the cefpodoxime proxetil susceptibility (including AmpC producers), or an infection control flag for ‘ESBL’, ‘CPO’ (carbapenemase-producing organisms), ‘MDR-Acinetobacter’ (carbapenem-resistant *Acinetobacter* spp.), and/or ‘MRO’ (MDR organisms, defined as Enterobacterales or *Pseudomonas* spp. with pan-β-lactam resistance not mediated by carbapenemase production). Positive *C. difficile* status was determined by the presence of a positive lateral flow test result for *C. difficile* toxin and/or glutamate dehydrogenase (GDH). Instances in which a patient’s status converted from negative in the 60 days prior to surgery to positive in the 60 days after surgery were recorded as newly identified infection/colonization. Procedures in which the patient’s status was already positive in the 60 days prior to surgery were excluded from analysis of the corresponding outcome.

### Covariates

Sex, age and American Society of Anesthesiologists (ASA) class at the time of surgery were extracted from Epic. BMI was calculated from the weight and height recorded in Epic at the time of surgery, and was categorized according to WHO definitions. For individuals aged <18 years at the time of surgery, Royal College of Paediatrics and Child Health (RCPCH) BMI charts were used to assign BMI category.^44^ Each of these variables was cross-referenced with data from the UKHSA SSISS database to minimize missing values. In instances of conflicting values, Epic data took precedence, unless there were plausibility concerns. Type of surgery, urgency of procedure, grade of operating surgeon and wound class were taken from the UKHSA SSISS database. Transplant status was determined by the presence of any of the following International Classification of Diseases (ICD10) codes in the patient’s Epic record prior to surgery or up to 30 days after surgery: Z94.0–Z94.6, Z94.8, Z94.9, T86.0–T86.9.

Medication data were extracted from Epic’s Medication Administration Record (MAR). Administered medications were filtered by name to include antibacterial agents, proton pump inhibitors (PPIs) and H2 receptor antagonists (H2RAs). MAR timestamps were used to determine the interval between medication administration and procedure onset. Antimicrobials were grouped according to class, as listed in the BNF.^45^ Antimicrobials were deemed to have comprised the prophylactic regimen if given at any time from 120 min prior to surgery until the recorded procedure end. Oral antimicrobials usually administered solely for medical prophylaxis, treating lower urinary infections or *C. difficile* treatment were excluded, as were other agents not routinely prescribed for surgical prophylaxis, such as oral macrolides and tetracyclines. Due to the large number of unique antimicrobial combinations administered in the peri-operative period, prophylactic regimens were categorized into commonly used backbone combinations: (i) β-lactam/β-lactamase inhibitor combination, or carbapenem; (ii) single-agent β-lactam with metronidazole, with or without gentamicin; (iii) gentamicin with metronidazole, in the absence of a β-lactam; (iv) ciprofloxacin with metronidazole, in the absence of gentamicin; and (v) an incomplete version of backbones (i)–(iv), or any other systemic antimicrobial. Broad-spectrum anaerobe cover was determined by the presence of a β-lactam/β-lactamase inhibitor combination, carbapenem, metronidazole or clindamycin in the regimen. Clindamycin was regarded as interchangeable with metronidazole for the purpose of regimen backbone categorization.

Timing of antimicrobial administration on the day of surgery was deemed appropriate for optimal prophylaxis if administered between 120 and 0 min pre-operatively. Cases in which antimicrobials were administered on the day of surgery, but outside of this window were categorized as having received them too early/late for optimum prophylaxis. Prophylactic gentamicin doses were calculated in mg/kg and categorized as lower dose (≤2 mg/kg) or higher dose (>2 mg/kg). Counts of total pre-operative and post-operative antimicrobial doses administered at CUH included all antibacterial classes.

### Statistical analysis

Baseline patient, admission, procedure and antimicrobial characteristics were compared across PAL status using Pearson’s chi-squared test for categorical variables and the Kruskal–Wallis test for continuous variables. Multivariable logistic regression was used to investigate the association between PALs and SSI, using Hosmer and Lemeshow’s purposeful selection.^46^ Briefly, all variables of predetermined clinical importance and those with *P* < 0.25 in univariable logistic regression were included in an initial multivariable model. Continuous variables for which there was no linear association with the logit of SSI were categorized into clinically relevant categories or according to the distribution of association with the logit of SSI. Variables with *P* > 0.05 in multivariable analysis were sequentially removed from the model, and the overall model’s impact was estimated using the Wald and likelihood-ratio tests. Each variable excluded from the initial multivariable model was then added to check for significant association in the presence of other model variables.

**Figure 1.**
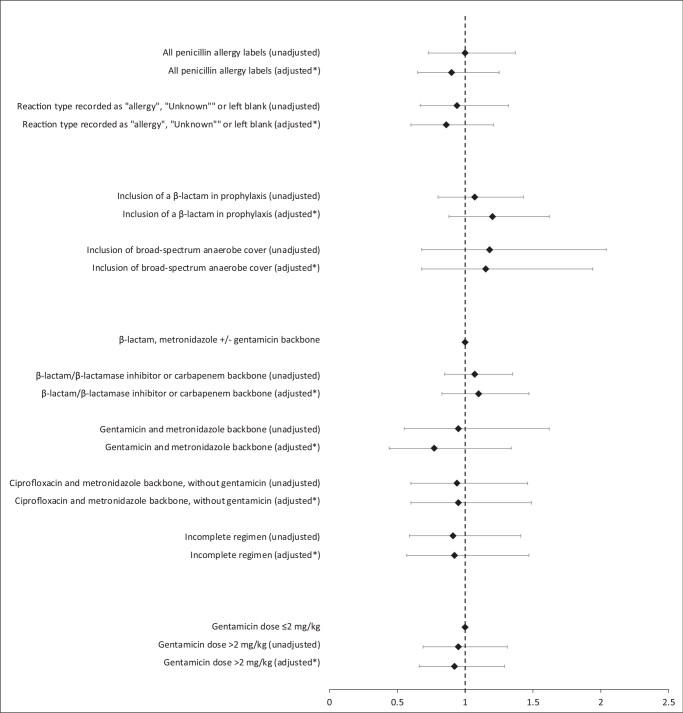
Unadjusted and aORs for SSI following operations undertaken in the presence of PALs and with different prophylactic antimicrobial regimens. *Multivariable adjustment for sex, age category, ASA class, type of surgery, duration of procedure, wound class and transplant status.

**Figure 2.**
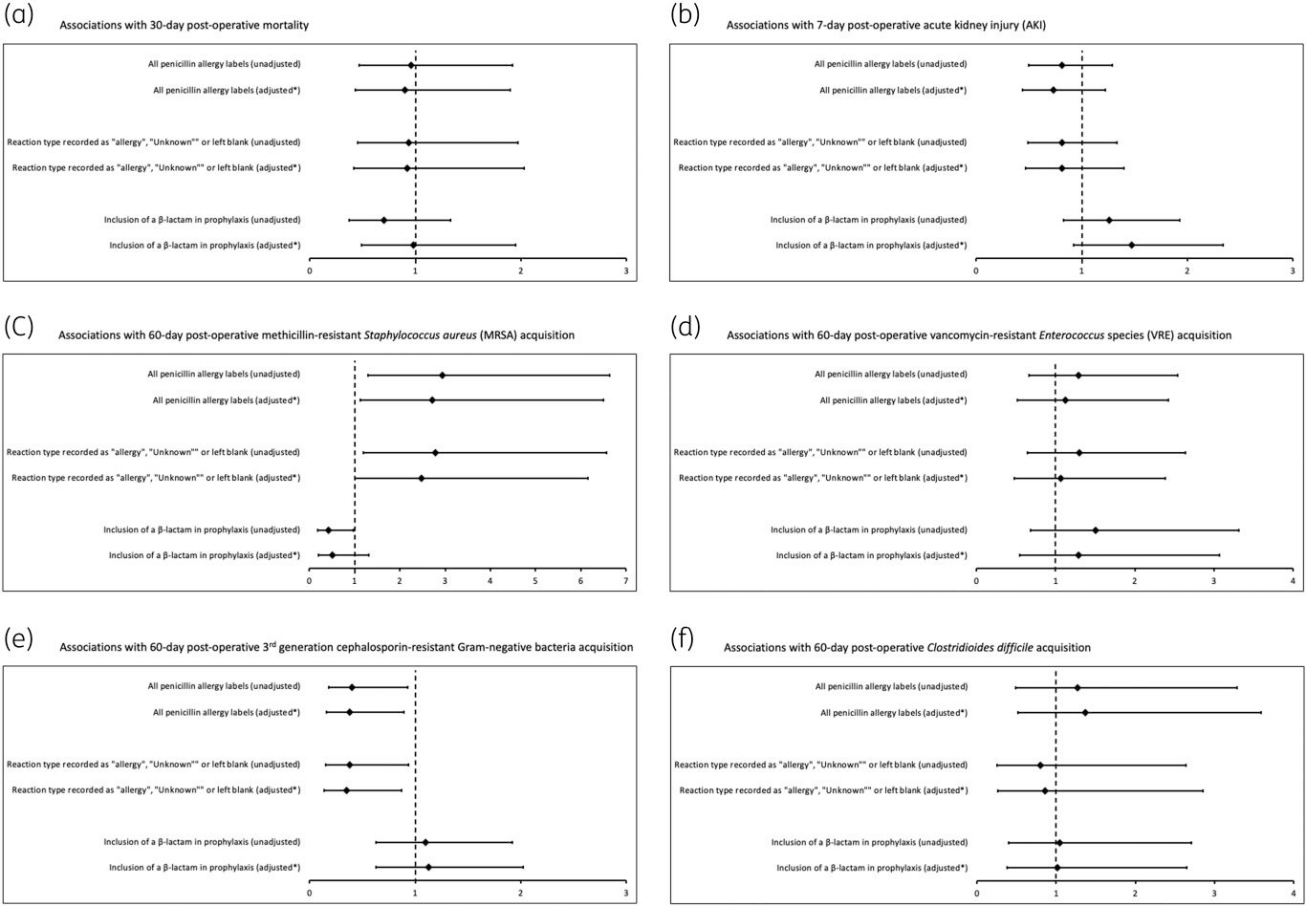
Unadjusted and aORs for 30 day mortality, 7 day post-operative AKI, 60 day post-operative MRSA infection/colonization acquisition, 60 day post-operative VRE species infection/colonization acquisition, 60 day post-operative third-generation cephalosporin-resistant Gram-negative infection/colonization acquisition and 60 day post-operative *C. difficile* infection/colonization acquisition. *(a) Multivariable adjustment: age category, ASA class and wound class; (b) multivariable adjustment: sex, BMI, ASA class, duration of procedure, 7 day pre-operative AKI and transplant status; (c) multivariable adjustment: BMI category, receipt of antimicrobials at CUH in the 6 months prior to surgery, and length of hospital stay; (d) multivariable adjustment: type of surgery, duration of procedure, receipt of antimicrobials at CUH in the 6 months prior to surgery, number of antimicrobial doses in the 28 days after surgery, length of hospital stay, and transplant status; (e) multivariable adjustment: duration of procedure, receipt of antimicrobials at CUH in the 6 months prior to surgery, number of antimicrobial doses in the 28 days after surgery, and length of hospital stay; (f) multivariable adjustment: number of antimicrobial doses in the 28 days after surgery and length of hospital stay. ‘Antimicrobials’ refers to antibacterial agents only.

This process was repeated separately for each secondary outcome to produce a series of unique multivariable models. In secondary analyses, each multivariable model was used to independently estimate adjusted ORs for: (i) PAL with reaction type recorded as ‘allergy’, ‘unknown/unable to establish’, or left blank; (ii) inclusion of a β-lactam agent in the prophylactic antimicrobial regimen; (iii) inclusion of broad-spectrum anaerobe cover; (iv) each of the different prophylactic antimicrobial regimen backbone categories; and (v) use of higher-dose gentamicin prophylaxis. In every model, individual operations were regarded as independent units of observation, including different procedures undertaken on the same patient. Adequacy of each model was assessed using the AUC. All statistical analyses were undertaken in Stata 17 software, and the forest plots for Figures 1 and 2 were created using Microsoft Excel.

### Ethics

This study was approved by the Health Research Authority and Health and Care Research Wales (REC number 21/EE/0292) and the CUH Research and Development Department (ref A096086). All patient identifiable data were pseudonymized by the hospital’s clinical informatics department prior to analysis.

## Results

The retrospective cohort included 3644 patients undergoing 4085 operations between January 2015 and December 2021, 461 of which were undertaken in the presence of a PAL (11.3%). For most procedures with a PAL present, the nature of reaction could be regarded as a probable or possible hypersensitivity reaction; the reaction type was recorded as ‘allergy’ in 182/461 (39.5%) of labels, ‘unknown/unable to establish’ in 27/461 (5.8%) and left blank in 205/461 (44.5%). Reaction type was recorded as ‘intolerance’ in 46/461 (10.0%) and ‘contraindication’ in 1/461 (0.2%). Severity of reaction was left unrecorded in 175/461 (38.0%) of cases and was recorded as ‘high’ in 78/461 (16.9%), ‘medium’ in 69/461 (15.0%), and ‘low’ in 139/461 (30.1%). Table 1 summarizes the patient demographic, hospital admission, surgical procedure and antimicrobial exposure characteristics of operations undertaken in the presence and absence of PALs.

**Table 1.** Baseline patient, admission, procedure and prophylactic antimicrobial characteristics

		All operations	PAL absent	PAL present	*P* value for comparison of the PAL-absent and PAL-present groups
Age at time of surgery (years)	Median (IQR)	62 (40–73)	61 (39–73)	66 (48–75)	0.001
	<1	326/4085 (8.0)	326/3634 (9.0)	0/461 (0.0)	<0.001
	1–5	85/4085 (2.1)	81/3634 (2.2)	4/461 (0.8)	
	6–25	230/4085 (5.6)	204/3634 (5.6)	26/461 (5.6)	
	26–45	542/4085 (13.3)	468/3634 (12.9)	74/461 (16.1)	
	46–65	1145/4085 (28.0)	1026/3634 (28.3)	119/461 (25.8)	
	66–85	1624/4085 (39.8)	1405/3634 (38.8)	219/461 (47.5)	
	>85	133/4085 (3.3)	114/3634 (3.2)	19/461 (4.1)	
Sex	Female	1933/4085 (47.3)	1635/3624 (45.1)	298/461 (64.6)	<0.001
	Male	2152/4085 (52.7)	1989/3624 (54.9)	163/461 (35.4)	
BMI at the time of surgery (kg/m^2^)	Median (IQR)	25.4 (21.9–29.0)	25.4 (22.0–28.9)	25.4 (21.9–29.2)	0.459
	Underweight	213/4085 (5.2)	185/3624 (5.1)	28/461 (6.0)	<0.001
	Healthy weight	1469/4085 (36.0)	1287/3624 (35.5)	182/461 (39.5)	
	Overweight	1223/4085 (29.9)	1082/3624 (29.9)	141/461 (30.6)	
	Obese	655/4085 (16.0)	560/3624 (15.4)	95/461 (20.6)	
	Severely obese	59/4085 (1.4)	55/3624 (1.5)	4/461 (0.9)	
	Missing	466/4085 (11.5)	455/3624 (12.6)	11/461 (2.4)	
Type of surgery	Large bowel	2313/4085 (56.6)	2041/3624 (56.3)	272/461 (59.0)	0.549
	Small bowel	1246/4085 (30.5)	1108/3624 (30.6)	138/461 (29.9)	
	Bile duct/liver/pancreas	334/4085 (8.2)	300/3624 (8.3)	34/461 (7.4)	
	Stomach/oesophagus	192/4085 (4.7)	175/3624 (4.8)	17/461 (3.7)	
Urgency of procedure	Elective	3789/4085 (92.8)	3361/3624 (92.7)	428/461 (92.8)	0.939
	Emergency	296/4085 (7.2)	263/3624 (7.3)	33/461 (7.2)	
Grade of operating surgeon	Consultant	3216/4085 (78.7)	2848/3624 (78.6)	368/461 (79.8)	0.257
	Registrar	739/4085 (18.1)	665/3624 (18.3)	74/461 (16.1)	
	Other	130/4085 (3.2)	111/3624 (3.1)	19/461 (4.1)	
Duration of operation (min)	Median (IQR)	165 (110–240)	165 (110–240)	170 (120–255)	0.395
	≤120	1243/4085 (30.4)	1110/3624 (30.6)	133/461 (28.9)	0.344
	121–240	1828/4085 (44.8)	1627/3624 (44.9)	201/461 (43.6)	
	>240	1014/4085 (24.8)	887/3624 (24.5)	127/461 (27.5)	
Wound class	Clean	95/4085 (2.4)	88/3624 (2.4)	7/461 (1.5)	0.286
	Clean-contaminated	2852/4085 (69.8)	2544/3624 (70.2)	308/461 (66.8)	
	Contaminated	581/4085 (14.2)	506/3624 (14.0)	75/461 (16.3)	
	Dirty	556/4085 (13.6)	485/3624 (13.4)	71/461 (15.4)	
	Missing	1/4085 (0.0)	1/3624 (0.0)	0/461 (0.0)	
ASA risk score	Class 1	278/4085 (6.8)	256/3624 (7.1)	22/461 (4.8)	0.002
	Class 2	1904/4085 (46.6)	1708/3624 (47.1)	196/461 (42.5)	
	Class 3	1417/4085 (34.7)	1227/3624 (33.9)	190/461 (41.2)	
	Class 4	344/4085 (8.4)	298/3624 (8.2)	46/461 (10.0)	
	Class 5	27/4085 (0.7)	26/3624 (0.7)	1/461 (0.2)	
	Missing	115/4085 (2.8)	109/3624 (3.0)	6/461 (1.3)	
History of solid organ or bone marrow transplant		118/4085 (2.9)	96/3624 (2.6)	22/461 (4.8)	0.010
AKI in the 7 days prior to surgery		60/4085 (1.5)	52/3624 (1.4)	8/461 (1.7)	0.613
Known infections or colonization in the 60 days prior to surgery	MRSA	35/4085 (0.9)	27/3624 (0.8)	8/461 (1.7)	0.027
	VRE	30/4085 (0.7)	25/3624 (0.7)	5/461 (1.1)	0.333
	AmpC/ESBL/CPE/MRO	79/4085 (1.9)	69/3624 (1.9)	10/461 (2.2)	0.663
	*C. difficile* (with or without detectable toxin production)	23/4085 (0.6)	18/3624 (0.5)	5/461 (1.1)	0.234
In receipt of antimicrobials at CUH in the 6 months prior to surgery		1330/4085 (32.6)	1188/3624 (32.8)	142/461 (30.8)	0.393
Number of antimicrobial doses at CUH in the 6 months prior to surgery, if given	Median (IQR)	9 (2–21)	9 (2–21)	8 (2–21)	0.922
	1–10	736/1330 (55.3)	655/1188 (55.1)	81/142 (57.0)	0.905
	11–20	244/1330 (18.4)	221/1188 (18.6)	23/142 (16.2)	
	21–30	145/1330 (10.9)	130/1188 (10.9)	15/142 (10.6)	
	>30	205/4085 (15.4)	182/1188 (15.3)	23/142 (16.2)	
Proton pump inhibitor or H2RA use	Present at the time of surgery	1408/4085 (34.5)	1220/3624 (33.7)	188/461 (40.8)	0.002
Length of pre-operative hospital stay (days)	Median (IQR)	0 (0–2)	0 (0–2)	0 (0–2)	0.595
Length of post-operative hospital stay (days)	Median (IQR)	9 (6–16)	9 (6–16)	9 (6–16)	0.449
Prophylactic antimicrobials administered (at any time from 2 h prior to surgery until the end of surgery)		3640/4085 (89.1)	3221/3624 (88.9)	419/461 (90.9)	0.192
Timing of antimicrobials on the day of surgery	Appropriate for optimal prophylaxis (<2 h pre-op until the start of surgery)	3296/4085 (80.7)	2917/3624 (80.5)	379/461 (82.2)	0.567
	Too early/late for optimal prophylaxis (2–24 h pre-op or intra-op only)	634/4085 (15.5)	566/3624 (15.6)	68/461 (14.8)	
	Not given at all	155/4085 (3.8)	141/3624 (3.9)	14/461 (3.0)	
Interval between pre-op antimicrobials and surgery (minutes)	Median (IQR)	20 (9–40)	21 (10–40)	16 (7–31)	<0.001
Repeat intraoperative dosing		552/3640 (15.2)	473/3640 (14.7)	79/419 (18.9)	0.025
B-lactam in regimen		3053/3640 (83.9)	2983/3221 (92.6)	70/419 (16.7)	<0.001
Broad spectrum anaerobe cover included in regimen		3473/3640 (95.4)	3111/3221 (96.6)	362/419 (86.4)	<0.001
Antimicrobial regimen backbone	β-Lactam/β-lactamase inhibitor combination, or carbapenem	1326/3640 (36.4)	1287/3221 (40.0)	39/419 (9.3)	<0.001
	β-Lactam and metronidazole, with or without gentamicin	1643/3640 (45.1)	1614/3221 (50.1)	29/419 (6.9)	
	Gentamicin and metronidazole	158/3640 (4.3)	84/3221 (2.6)	74/419 (17.7)	
	Ciprofloxacin and metronidazole (without gentamicin)	248/3640 (6.8)	49/3221 (1.5)	199/419 (47.5)	
	Incomplete regimen	265/3640 (7.3)	187/3221 (5.8)	78/419 (18.6)	
Prophylactic gentamicin dose if given (mg/kg)	Median (IQR)	1.74 (1.48–2.05)	1.74 (1.48–2.05)	1.79 (1.56–2.02)	0.388
	≤2	1341/1851 (72.5)	1260/1742 (72.3)	81/109 (74.3)	0.653
	>2	510/1851 (27.6)	482/1742 (27.7)	28/109 (25.7)	
In receipt of antimicrobials at CUH at any time from the end of surgery until 28 days post-surgery		2661/4085 (65.1)	2372/3624 (65.5)	172/461 (62.7)	0.241
Number of antimicrobial doses at CUH in the 28 days after surgery, if given	Median (IQR)	18 (10–25)	18 (10–25)	18 (9–25)	0.881
	≤10	701/2661 (26.3)	615/2372 (25.9)	86/289 (29.7)	0.354
	11–20	863/2661 (32.4)	778/2372 (32.8)	85/289 (29.4)	
	21–30	603/2661 (22.7)	543/2372 (22.9)	60/289 (20.8)	
	>30	494/2661 (18.6)	436/2372 (18.4)	58/289 (20.1)	

Values are given as *n*/*N* (%) unless otherwise stated. ‘Antimicrobials’ refers to antibacterial agents only.

Female sex was more common in operations undertaken in the presence of a PAL (64.6% versus 45.1%; *P* < 0.001), as was older age (median 66 versus 61 years; *P* < 0.001), history of solid organ or bone marrow transplant (4.8% versus 2.6%; *P* = 0.010), receipt of PPI or H2RA therapy (40.8% versus 33.7%; *P* = 0.002) and known pre-operative MRSA infection/colonization (1.7% versus 0.8%; *P* = 0.027). ASA class distributions also differed significantly between groups (*P* = 0.002). A significant difference in the distribution of BMI categories was observed (*P* = 0.001), but this was driven by the proportion of operations for which BMI data were missing (2.4% versus 12.6%).

There was no significant difference in the proportion of operations in which antimicrobial prophylaxis was used, nor that had antimicrobials administered within the optimal prophylaxis time period. However, the median interval between the latest pre-operative antimicrobial dose and the start of surgery was significantly shorter for procedures in which patients had a PAL (16 min versus 21 min; *P* < 0.001), and repeat intra-operative doses were more likely (18.9% versus 14.7%; *P* = 0.025). Benzylpenicillin, metronidazole and gentamicin was the most commonly observed prophylaxis regimen, used in 1580/3640 (43.4%) procedures (Table S2). Although exclusively non-β-lactam agents were used in most instances of PAL, β-lactams were administered in 15.2% of PAL cases; penicillins in 9.3%, cephalosporins in 1.3% and carbapenems in 4.6% (Table 2).

**Table 2.** Use of β-lactam agents as peri-operative prophylaxis in the presence of penicillin allergy labels

	All PALs	PALs with reaction type recorded as ‘allergy’, ‘unknown/unable to establish’ or left blank	PALs with reaction type recorded as ‘allergy’, ‘unknown/unable to establish’ or left blank, and severity recorded as ‘high’
No prophylactic antimicrobials given	42/461 (9.1)	41/414 (9.9)	7/77 (9.1)
No β-lactams included in regimen	349/461 (75.7)	318/414 (76.8)	62/77 (80.5)
Penicillin included in regimen	43/461 (9.3)	31/414 (7.5)	3/77 (3.9)
* *Benzylpenicillin	25/461 (5.4)	17/414 (4.1)	3/77 (3.9)
* *Co-amoxiclav	13/461 (2.8)	11/414 (2.7)	0/77 (0.0)
* *Piperacillin/tazobactam	5/461 (1.1)	3/414 (0.7)	0/77 (0.0)
Cephalosporin included in regimen	6/461 (1.3)	6/414 (1.4)	0/77 (0.0)
* *Ceftriaxone	5/461 (1.1)	5/414 (1.2)	0/77 (0.0)
* *Ceftazidime	1/461 (0.2)	1/414 (0.2)	0/77 (0.0)
Carbapenem included in regimen^a^	21/461 (4.6)	18/414 (4.3)	5/77 (6.5)

Values are given as *n*/*N* (%) unless otherwise stated.

^a^Meropenem used in all instances of carbapenem use.

SSIs were detected after 435/4085 procedures (10.7%), with no significant variation in incidence by calendar year or 3 month observation period. Sex, age category, ASA class, surgery type, procedure duration, wound class and transplant status were associated with SSI in multivariable analysis (AUC 0.6821) (Table S3). In univariable and multivariable analysis, the presence of a PAL was not found to be associated with SSI, nor was the avoidance of β-lactam-based prophylactic regimens (Figure 1 and Table S4). Higher dosing of gentamicin prophylaxis was not found to have a significant impact on odds of SSI.

Factors independently associated with each secondary outcome are summarized in Tables S5–S10 (AUC shown for each model as table footnotes). In multivariable analysis, neither the presence of a PAL, nor avoidance of β-lactam-based prophylaxis were found to be associated with 30 day post-operative mortality, 7 day post-operative AKI, or newly identified post-operative VRE or *C. difficile* infection/colonization (Figure 2, Tables S11–S14). PALs were associated with significantly higher odds of newly identified post-operative MRSA infection/colonization (aOR 2.82; 95% CI 1.18–6.75) (Figure 2, Table S15). Conversely, odds of newly identified post-operative infection/colonization with Gram-negative bacteria resistant to third-generation cephalosporins were found to be significantly lower when PALs were present (aOR 0.37; 95% CI 0.16–0.87) (Figure 2, Table S16).

When compared with the use of a β-lactam and metronidazole prophylactic regimen backbone (with or without gentamicin), 30 day post-operative mortality was found to be significantly more likely with operations that used a β-lactam/β-lactamase combination or carbapenem backbone (aOR 3.05; 95% CI 1.29–7.25) or an incomplete regimen (aOR 3.03; 95% CI 1.03–8.93) (Table S11). This observation was unaffected by adding adjustment for the number of uninterrupted days of peri-operative antimicrobial treatment or transplant status. There was an increase in likelihood of newly identified post-operative *C. difficile* infection/colonization when a ciprofloxacin and metronidazole backbone had been used, but this association was not statistically significant (aOR 3.20; 95% CI 0.91–11.24) (Table S14).

## Discussion

Recorded allergy to penicillin has been associated with a wide range of adverse clinical outcomes, including in recent reports of increased SSI risk amongst surgical patients in the USA, Canada and France. In this large retrospective UK cohort of gastrointestinal surgery patients, we observed no evidence of association between PALs and SSI, highlighting important international variation in the clinical impact of PALs on surgical patients. Prevalence of PALs was high, in keeping with previous reports from our own hospital and internationally.^14,15^ With penicillin allergy de-labelling programmes becoming increasingly common across the world,^47^ consideration should be given to the local epidemiology of clinical burden from PALs to ensure de-labelling interventions are targeted towards patient groups with the most benefit to gain.

Our findings are inconsistent with those of one Canadian and two US studies to have considered the effect of PALs on SSI in mixed cohorts that included gastrointestinal procedure types, each of which reported adjusted ORs in the range 1.51 to 2.02. Although a reasonably high proportion of operations undertaken in the presence of a PAL involved the use of β-lactam-based prophylaxis in our study, this is consistent with the observations of others^33,38^ and we found no evidence of association between non-β-lactam-based prophylaxis and odds of SSI to suggest that this was a confounding factor. Other reports of association between PALs and SSI are predominantly from the USA, with only one additional published study from France. These studies were all restricted to patients undergoing non-gastrointestinal surgery,^36–41^ and this difference in case mix could partially explain the disagreement with our findings. Of note, the only previously published study to examine the effect of PALs exclusively in gastrointestinal surgical patients found no evidence of association with SSI.^48^ Absence of observed association has also been reported from two cohorts of arthroplasty patients and a small cohort of neurosurgical patients in the USA.^49–51^

Other important differences between our study and those to have described significant association between PALs and SSI are inherent differences in the epidemiology of AMR^52^ and important variation in antimicrobial prophylaxis guidelines.^1,53,54^ Joint guidance from the American Society of Health-System Pharmacists (ASHP), IDSA, the Society for Healthcare Epidemiology of America (SHEA) and the Surgical Infection Society (SIS) recommends specific antimicrobial regimens for each of the most common procedure types, with cefazolin featuring prominently for patients without a PAL and clindamycin for those labelled allergic.^53^ In contrast, guidelines from the UK and Europe do not stipulate any preferred antimicrobial regimens.^1,54^ As a result, a large amount of heterogeneity exists in the composition of local antimicrobial prophylaxis protocols in the UK, and it is uncommon for cefazolin to be recommended as a first-line agent. We observed no instances of cefazolin use and only two instances of clindamycin use in our cohort. Importantly, clindamycin has been consistently identified as a suboptimal prophylactic agent in patients with a PAL in prior studies.^33,40,41^

Due to its observational nature, our study did not involve routine rectal swab or faecal sampling to systematically assess the prevalence of AMR colonization amongst the study cohort. However, we know from local AMR screening surveillance data that the hospital-wide proportion of positive ESBL, CPE and VRE screening samples varied significantly during the observation period of this study (highest ESBL screen positivity rate 6.3% in 2017, lowest 2.8% in 2019; highest CPE screen positivity rate 1.7% in 2018, lowest 1.3% in 2020; highest VRE screen positivity rate 21.2% in 2015, lowest 14.6% in 2017). The proportion of positive MRSA screening samples was relatively consistent, ranging from 1.3% in 2015 to 1.5% in 2018. Interestingly, we did not observe significant variation in rates of SSI over time during the observation period, suggesting that fluctuating prevalence of ESBL/CPE/VRE colonization in the patient populations captured by the hospital’s routine screening protocols did not directly impact SSI frequency in our study cohort.

The presence of PALs was associated with increased likelihood of newly identified MRSA in the 60 days after surgery in our study, which is consistent with both the relative increase in frequency of pre-operative MRSA infection/colonization in the PAL group and prior published literature on the association between PALs and MRSA infection/colonization in wider healthcare populations.^22,28^ There was no evidence of association between any particular prophylactic regimen and post-operative MRSA acquisition, indicating that antimicrobial exposures outside of the immediate peri-operative period are the likely mediators of MRSA risk. We also observed an apparent protective effect of PALs on the likelihood of 60 day post-operative acquisition of third-generation cephalosporin-resistant Gram-negative bacteria, which has not been previously reported. An underlying causal relationship is plausible because patients with a PAL are less likely to have been exposed to the selection pressures of cephalosporin and β-lactam/β-lactamase inhibitor combination exposures during their hospital admission.

The use of a β-lactam/β-lactamase inhibitor combination or carbapenem backbone was associated with higher odds of 30 day post-operative mortality in our cohort, when compared with the use of a narrow-spectrum β-lactam in combination with metronidazole, with or without gentamicin. However, this observation may be confounded by differences in perceived levels of patient or procedural risk not accounted for by our covariate measures, as many of the antimicrobials in the β-lactam/β-lactamase inhibitor combination group were agents usually reserved for treating the most high-risk patients and infections at our hospital. Although the observed trend towards increased likelihood of post-operative *C. difficile* infection/colonization acquisition with the use of ciprofloxacin plus metronidazole backbone prophylaxis was not statistically significant, this may be explained by the relatively low number of *C. difficile* infection/colonization events in the study. Fluoroquinolone treatment is well recognized as a risk factor for *C. difficile* infection^55^ and the potential impact of ciprofloxacin use in surgical prophylaxis deserves further research. Similarly, post-operative mortality within 30 days of surgery is relatively uncommon, so our analysis of this outcome measure is hampered by low statistical power.

This study has limitations. First, despite active inpatient SSI surveillance up to 30 days in the prospective surveillance programme, there was no process of active surveillance beyond hospital discharge, meaning any SSI occurring within 30 days of surgery in outpatient settings will have remained undetected. However, there is no reason to believe that these will have been unevenly distributed between patients with and without PALs. Antimicrobial administration in the 28 days after surgery was relatively high in this cohort, but this did not differ between PAL groups. Data were also unavailable for some potentially important variables; pre-operative skin preparation, intraoperative control of body temperature, glycaemia and oxygenation, and the use of the WHO surgical safety checklist have all been suggested as important factors in reducing SSI risk.^56,57^ We were also unable to capture microbiology data from external laboratories, which may have been relevant to our secondary outcome measures in a small number of cases. In addition, the heterogeneity in UK antimicrobial prophylaxis guidance means that our findings may not be generalizable to the wider UK population. Indeed, CUH is an outlier in recommending a benzylpenicillin, metronidazole and gentamicin combination regimen as first-line prophylaxis for colorectal procedures. However, this point merely serves to emphasize the importance of local epidemiology and antimicrobial prescribing when considering the adverse clinical impacts of PALs in particular patient groups.

Our findings suggest that the association between PALs and SSI is not absolute and likely depends on the type of surgery undertaken and locally determined factors, such as the epidemiology of infecting pathogens and types of antimicrobial regimens in routine use. Further work should focus on differentiating these mediating factors and analysing the potential magnitude of intended benefits of penicillin allergy de-labelling initiatives in specific patient groups.

## Supplementary Material

dlae022_Supplementary_Data

## Data Availability

The data that support the findings of this study are available from the authors upon reasonable request, with prior permission from Cambridge University Hospitals NHS Foundation Trust and appropriate Research Ethics Committee approval.

## Supplementary data

Tables S1 to S16 are available as Supplementary data at *JAC-AMR* Online.
