# Supplementary material for: Impact of penicillin allergy labels on surgical site infections in a large UK cohort of gastrointestinal surgery patients
Source: JAC Antimicrob Resist. 2024 Feb 16;6(1):dlae022. doi: 10.1093/jacamr/dlae022 (PMC10873540; doi:10.1093/jacamr/dlae022)
Supplement: dlae022_Supplementary_Data [file dlae022_supplementary_data.docx]

**Table S1. Peri-operative prophylactic antimicrobial guidelines at Cambridge University Hospitals NHS Foundation Trust (CUH)**

| **Procedure** | **First line antimicrobial prophylaxis recommendation** | **Antimicrobial prophylaxis recommendation in patients with penicillin allergy** |
| --- | --- | --- |
| ‘Uncomplicated’ gastrectomy,  oesophagectomy, fundoplications (no active infection) | Single dose co-amoxiclav† 1.2g iv  If MRSA risk, add a single dose of teicoplanin 10mg/kg (max 800mg) iv | Single dose teicoplanin 10mg/kg (max 800mg) iv + gentamicin 1.5mg/kg iv |
| ‘Complicated’ gastrectomy, oesophagectomy, fundoplications (e.g. gastric stasis, perforation, spillage at operation) | Co-amoxiclav 1.2g† iv 8-hourly  If MRSA risk, add teicoplanin 10mg/kg (max 800mg) iv 24-hourly  Duration: up to 48 hours | Penicillin allergy +/- MRSA risk  teicoplanin 10mg/kg (max 800mg) iv 24-hourly + gentamicin 5mg/kg (max 480mg) iv 24-hourly + metronidazole 500mg iv 8-hourly  Duration: up to 48 hours |
| Open hernia repair (not laparoscopic) | Single dose flucloxacillin 1g | Single dose teicoplanin 10mg/kg (max 800mg) iv |
| Laparascopic cholecystectomy (only if gall bladder significantly inflamed) | Single dose co-amoxiclav 1.2g iv  If MRSA risk, add a single dose of teicoplanin 10mg/kg (max 800mg) iv | Single dose: teicoplanin 10mg/kg (max 800mg) iv + gentamicin 1.5mg/kg iv |
| ‘Uncomplicated’ appendicectomy | Single dose co-amoxiclav 1.2g iv  If MRSA risk, add a single dose of teicoplanin 10mg/kg (max 800mg) iv | Single dose gentamicin 1.5mg/kg iv + metronidazole 500mg iv  If MRSA risk, add a single dose of teicoplanin 10mg/kg (max 800mg) iv |
| ‘Complicated’ appendicectomy (perforated, gangrenous, widespread peritonitis/unwell with significant peritoneal contamination) | Piperacillin-tazobactam† 4.5g IV 8-  Hourly  If MRSA risk, add teicoplanin 10mg/kg (max 800mg) iv 24-hourly  Duration: up to 48 hours, then review the need for continuation | Gentamicin 5mg/kg (max 480mg) iv 24 hourly + metronidazole 500mg iv 8-hourly  If MRSA risk, add  teicoplanin 10mg/kg (max 800mg) iv 24-hourly  Duration: up to 48 hours, then review the need for continuation |
| ‘Minor’ hepatobiliary procedures e.g. laparoscopic biliary surgery in high risk patients, and minor hepatic resection | Single dose co-amoxiclav 1.2g iv  If MRSA risk, add a single dose of teicoplanin 10mg/kg (max 800mg) iv | Single dose gentamicin 1.5mg/kg iv + metronidazole 500mg iv  If MRSA risk, add a single dose of teicoplanin 10mg/kg (max 800mg) iv |
| ‘Major’ hepatobiliary procedures e.g. pancreatectomy and major liver resection | Co-amoxiclav 1.2g† iv 8-hourly  If MRSA risk, add a single dose of teicoplanin 10mg/kg (max 800mg) iv  Duration: 24 hours | Single dose gentamicin 5mg/kg iv (max 480mg) + metronidazole 500mg iv 8-hourly  If MRSA risk, add a single dose of teicoplanin 10mg/kg (max 800mg) iv  Duration: 24 hours |
| ‘Complicated’ open biliary surgery and resections including Whipples, or hepatic with biliary stent or drain | Piperacillin-tazobactam† 4.5g iv 8-hourly  If MRSA risk, add a single dose of teicoplanin 10mg/kg (max 800mg) iv  Duration: 24 hours  , then review the need for continuation | A single dose of teicoplanin 10mg/kg (max 800mg) iv + ciprofloxacin 400mg iv 12-hourly + metronidazole 500mg iv 8-hourly  Duration: 24 hours   , then review the need for continuation |
| Elective colectomy, resections of colon and rectum for carcinoma &  resections in inflammatory bowel disease | Single dose:  Benzylpenicillin† 1.2g iv + gentamicin 1.5mg/kg iv + metronidazole 500mg iv  If MRSA risk, use the regimen recommended in penicillin allergy | Single dose:  Teicoplanin 10mg/kg (max 800mg) iv  Gentamicin 1.5mg/kg iv +  Metronidazole 500mg iv |
| Emergency colectomy (eg AP resection, spillage of gut contents at operation etc.) | Benzylpenicillin† 1.2g iv 6-hourly + gentamicin* 5mg/kg (max  480mg) iv 24-hourly + metronidazole 500mg iv 8-hourly  If MRSA risk, use the regimen recommended in penicillin allergy  Duration: up to 48 hours  *If renal impairment of creatinine clearance <20ml/min – give ciprofloxacin 400mg iv 12-hourly instead of gentamicin | A single dose of teicoplanin 10mg/kg (max 800mg) iv 24-hourly + gentamicin* 5mg/kg (max 480mg) iv 24-hourly + metronidazole 500mg iv 8-hourly  Duration: up to 48 hours  *If renal impairment of creatinine clearance <20ml/min – give ciprofloxacin 400mg iv 12-hourly instead of gentamicin |
| Liver transplant | Piperacillin-tazobactam† 4.5g iv 8-hourly  If MRSA risk, add vancomycin 1g iv  Duration: 48 hours | Ciprofloxacin 400 mg iv 12-hourly + metronidazole 500 mg iv 8-hourly + vancomycin iv  Duration 48 hours |
| Renal transplant | Co-amoxiclav 1.2g iv at induction and again 8 hours later | Vancomycin 1g iv + ciprofloxacin  400mg iv to be given at induction only |
| Combined kidney and pancreas transplant | Piperacillin-tazobactam† 4.5g iv 8-hourly  If MRSA risk, add vancomycin 1g iv at induction only  Duration: 48 hours | If non-severe allergy: meropenem 500 mg iv  If severe allergy: vancomycin 1g iv + ciprofloxacin 400mg iv + metronidazole 400mg iv  Duration: 48 hours |
| Small bowel or multivisceral transplant | Piperacillin-tazobactam† 4.5g iv 8-hourly  If MRSA risk, add teicoplanin 10 mg/kg iv at induction only  Duration: 5 days | Meropenem 1g iv 8-hourly  If MRSA risk, add teicoplanin 10 mg/kg iv at induction only  Duration: 5 days |

†Give a repeated dose intraoperatively if the duration of surgery exceeds 4 hours, or after fluid replacement due to >1.5L of intraoperative blood loss

**Table S2. Antimicrobials administered as peri-operative prophylaxis**

| **β-lactam, metronidazole +/- gentamicin as regimen backbone** | **1,643/3,640 (45.1%)** |
| --- | --- |
| *Benzylpenicillin, gentamicin and metronidazole* | *1,580/1,643 (96.2%)* |
| *Ceftriaxone and metronidazole* | *38/1,643 (2.3%)* |
| *Flucloxacillin, gentamicin and metronidazole* | *8/1,643 (0.5%)* |
| *Amoxicillin, gentamicin and metronidazole* | *6/1,643 (0.4%)* |
| *Ceftriaxone, gentamicin and metronidazole* | *5/1,643 (0.3%)* |
| *Ceftazidime and metronidazole* | *2/1,643 (0.1%)* |
| *Flucloxacillin, benzylpenicillin, gentamicin and metronidazole* | *2/1,643 (0.1%)* |
| *Teicoplanin, benzylpenicillin, gentamicin and metronidazole* | *1/1,643 (0.1%)* |
| *Ciprofloxacin, benzylpenicillin, gentamicin and metronidazole* | *1/1,643 (0.1%)* |
| **β-lactam with β-lactamase inhibitor, or carbapenem as regimen backbone** | **1,326/3,640 (32.5%)** |
| *Co-amoxiclav* | *751/1,326 (56.6%)* |
| *Piperacillin-tazobactam* | *268/1,326 (20.2%)* |
| *Co-amoxiclav and gentamicin* | *68/1,326 (5.1%%)* |
| *Meropenem* | *67/1,326 (5.1%%)* |
| *Co-amoxiclav, gentamicin and metronidazole* | *41/1,326 (3.1%)* |
| *Co-amoxiclav and metronidazole* | *27/1,326 (2.0%)* |
| *Piperacillin-tazobactam and gentamicin* | *24/1,326 (1.8%)* |
| *Piperacillin-tazobactam and metronidazole* | *15/1,326 (1.1%)* |
| *Co-amoxiclav, benzylpenicillin, gentamicin and metronidazole* | *13/1,326 (1.0%)* |
| *Piperacillin-tazobactam and co-amoxiclav* | *11/1,326 (0.8%)* |
| *Meropenem and vancomycin* | *6/1,326 (0.5%)* |
| *Piperacillin-tazobactam, gentamicin and metronidazole* | *6/1,326 (0.5%)* |
| *Meropenem and metronidazole* | *4/1,326 (0.3%)* |
| *Piperacillin-tazobactam, vancomycin and metronidazole* | *3/1,326 (0.2%)* |
| *Piperacillin-tazobactam, benzylpenicillin, gentamicin and metronidazole* | *3/1,326 (0.2%)* |
| *Piperacillin-tazobactam and vancomycin* | *2/1,326 (0.2%)* |
| *Co-amoxiclav and vancomycin* | *2/1,326 (0.2%)* |
| *Co-amoxiclav and ciprofloxacin* | *2/1,326 (0.2%)* |
| *Meropenem and teicoplanin* | *1/1,326 (0.1%)* |
| *Meropenem and piperacillin-tazobactam* | *1/1,326 (0.1%)* |
| *Meropenem, co-trimoxazole and gentamicin* | *1/1,326 (0.1%)* |
| *Co-amoxiclav, vancomycin and metronidazole* | *1/1,326 (0.1%)* |
| *Piperacillin-tazobactam, co-amoxiclav and metronidazole* | *1/1,326 (0.1%)* |
| *Meropenem and co-amoxiclav* | *1/1,326 (0.1%)* |
| *Piperacillin-tazobactam, co-amoxiclav and gentamicin* | *1/1,326 (0.1%)* |
| *Co-amoxiclav and flucloxacillin* | *1/1,326 (0.1%)* |
| *Co-amoxiclav, flucloxacillin and gentamicin* | *1/1,326 (0.1%)* |
| *Piperacillin-tazobactam and ceftriaxone* | *1/1,326 (0.1%)* |
| *Meropenem, benzylpenicillin and gentamicin* | *1/1,326 (0.1%)* |
| *Piperacillin-tazobactam and flucloxacillin* | *1/1,326 (0.1%)* |
| *Co-amoxiclav, amoxicillin, gentamicin and metronidazole* | *1/1,326 (0.1%)* |
| **Gentamicin and metronidazole as regimen backbone** | **158/3,640 (4.3%)** |
| *Gentamicin, metronidazole and teicoplanin* | *60/158 (38.0%)* |
| *Gentamicin and metronidazole* | *47/158 (29.8)* |
| *Gentamicin, metronidazole and vancomycin* | *27/158 (17.1%)* |
| *Gentamicin, metronidazole and ciprofloxacin* | *22/158 (13.9%)* |
| *Gentamicin, clindamycin* and vancomycin* | *1/158 (0.6%)* |
| *Gentamicin, metronidazole, ciprofloxacin and vancomycin* | *1/158 (0.6%)* |
| **Ciprofloxacin and metronidazole as regimen backbone (without gentamicin)** | **248/3,640 (6.8%)** |
| *Ciprofloxacin and metronidazole* | *223/248 (89.9%)* |
| *Ciprofloxacin, metronidazole and vancomycin* | *2/248 (8.5%)* |
| *Ciprofloxacin, metronidazole and benzylpenicillin* | *2/248 (0.8%)* |
| *Ciprofloxacin, metronidazole and teicoplanin* | *1/248 (0.4%)* |
| *Ciprofloxacin and clindamycin** | *1/248 (0.4%)* |
| **Incomplete regimen** | **265/3,640 (7.3%)** |
| *Metronidazole* | *60/265 (22.6%)* |
| *Gentamicin* | *39/265 (14.7%)* |
| *Gentamicin and benzylpenicillin* | *31/265 (11.7%)* |
| *Ciprofloxacin* | *27/265 (10.2%)* |
| *Benzylpenicillin and metronidazole* | *27/265 (10.2%)* |
| *Vancomycin* | *17/265 (6.4%)* |
| *Benzylpenicillin* | *14/265 (5.3%)* |
| *Ciprofloxacin and vancomycin* | *9/265 (3.4%)* |
| *Metronidazole and vancomycin* | *7/265 (2.6%)* |
| *Gentamicin and ciprofloxacin* | *5/265 (1.9%)* |
| *Gentamicin and vancomycin* | *4/265 (1.5%)* |
| *Ceftriaxone* | *4/265 (1.5%)* |
| *Teicoplanin* | *3/265 (1.1%)* |
| *Gentamicin and teicoplanin* | *2/265 (0.8%)* |
| *Gentamicin and flucloxacillin* | *2/265 (0.8%)* |
| *Co-trimoxazole* | *2/265 (0.8%)* |
| *Ciprofloxacin and teicoplanin* | *2/265 (0.8%)* |
| *Gentamicin, ciprofloxacin and vancomycin* | *2/265 (0.8%)* |
| *Amoxicillin and metronidazole* | *2/265 (0.8%)* |
| *Tigecycline* | *1/265 (0.4%)* |
| *Metronidazole and teicoplanin* | *1/265 (0.4%)* |
| *Metronidazole, teicoplanin and vancomycin* | *1/265 (0.4%)* |
| *Co-trimoxazole and vancomycin* | *1/265 (0.4%)* |
| *Ceftazidime and vancomycin* | *1/265 (0.4%)* |
| *Amoxicillin and gentamicin* | *1/265 (0.4%)* |

*Clindamycin included in the place of metronidazole in the regimen backbone categorisation. Includes all systemic antimicrobials with the potential for perioperative prophylaxis that were administered at any time from 2 hours prior to the onset of surgery until the recorded procedure end.

**Table S3. Factors associated with odds of surgical site infection**

| Variable | Univariable logistic regression | | | Multivariable logistic regression  (Adjustment for all other variables in table) | | |
| --- | --- | --- | --- | --- | --- | --- |
|  | Odds ratio | 95% confidence interval | P-value | Adjusted odds ratio | 95% confidence interval | P-value |
| Male sex | 1.24 | 1.02-1.51 | 0.035 | 1.23 | 1.00-1.52 | 0.047 |
| Age <1 | *Reference category* | | | *Reference category* | | |
| Age 1-5 years | 0.60 | 0.17-2.05 | 0.407 | 0.64 | 0.18-2.25 | 0.485 |
| Age 6-25 years | 0.97 | 0.47-2.00 | 0.93 | 1.04 | 0.49-2.23 | 0.911 |
| Age 26-45 years | 1.82 | 1.06-3.13 | 0.029 | 1.68 | 0.95-2.97 | 0.074 |
| Age 46-65 years | 2.32 | 1.42-3.81 | 0.001 | 2.25 | 1.33-3.80 | 0.002 |
| Age 66-85 years | 2.01 | 1.24-3.28 | 0.005 | 1.79 | 1.07-3.01 | 0.027 |
| Age >85 years | 3.03 | 1.57-5.85 | 0.001 | 2.69 | 1.35-5.37 | 0.005 |
| ASA class 1 | *Reference category* | | | *Reference category* | | |
| ASA class 2 | 1.23 | 0.75-2.02 | 0.404 | 0.95 | 0.57-1.58 | 0.832 |
| ASA class 3 | 2.14 | 1.31-2.49 | 0.002 | 1.60 | 0.95-2.67 | 0.077 |
| ASA class 4 | 2.43 | 1.40-4.21 | 0.002 | 1.80 | 1.00-3.23 | 0.049 |
| ASA class 5 | 3.10 | 1.06-9.10 | 0.040 | 2.22 | 0.72-6.87 | 0.165 |
| Large bowel surgery | *Reference category* | | | *Reference category* | | |
| Small bowel surgery | 0.82 | 0.56-1.19 | 0.290 | 0.82 | 0.54-1.27 | 0.376 |
| Bile duct / liver/ pancreas surgery | 0.23 | 0.10-0.53 | 0.001 | 0.37 | 0.16-0.84 | 0.018 |
| Stomach / oesophagus surgery | 0.72 | 0.57-0.90 | 0.005 | 0.70 | 0.54-0.91 | 0.008 |
| Operation duration ≤120 minutes | *Reference category* | | | *Reference category* | | |
| Operation duration 121-240 minutes | 1.37 | 1.06-1.77 | 0.015 | 1.35 | 1.03-1.77 | 0.032 |
| Operation duration >240 minutes | 1.95 | 1.49-2.56 | <0.001 | 2.12 | 1.57-2.87 | <0.001 |
| Dirty wound | *Reference category* | | | *Reference category* | | |
| Contaminated wound | 0.53 | 0.38-0.74 | <0.001 | 0.74 | 0.52-1.05 | 0.090 |
| Clean-contaminated wound | 0.40 | 0.31-0.51 | <0.001 | 0.39 | 0.30-0.51 | <0.001 |
| Clean wound | 0.23 | 0.09-0.57 | 0.002 | 0.19 | 0.07-0.53 | 0.001 |
| History of SOT or BMT | 0.77 | 0.40-1.49 | 0.438 | 0.49 | 0.25-0.97 | 0.040 |

ASA; American Society of Anaesthesiologists. SOT; solid organ transplant. BMT; bone marrow transplant. AUC for the multivariable model: 0.6821.

**Table S4. Unadjusted and adjusted odds ratios for surgical site infection following operations undertaken in the presence of penicillin allergy labels and with different prophylactic antimicrobial regimens**

| Variable | Univariable logistic regression | | | Multivariable logistic regression* | | |
| --- | --- | --- | --- | --- | --- | --- |
|  | Odds ratio | 95% confidence interval | P-value | Adjusted odds ratio | 95% confidence interval | P-value |
| Penicillin allergy label (any) | 1.00 | 0.73-1.37 | 0.988 | 0.90 | 0.65-1.25 | 0.540 |
| Penicillin allergy label with reaction type recorded as ‘Allergy’, ‘Unknown/unable to establish’ or left blank | 0.94 | 0.67-1.32 | 0.726 | 0.86 | 0.60-1.21 | 0.385 |
| β-lactam included in regimen | 1.07 | 0.80-1.43 | 0.640 | 1.20 | 0.88-1.62 | 0.249 |
| Broad-spectrum anaerobe cover included in regimen | 1.15 | 0.68-1.94 | 0.612 | 1.18 | 0.68-2.04 | 0.548 |
| β-lactam, metronidazole +/- gentamicin as regimen backbone | *Reference category* | | | *Reference category* | | |
| β-lactam with β-lactamase inhibitor, or carbapenem as regimen backbone | 1.07 | 0.85-1.35 | 0.566 | 1.10 | 0.83-1.47 | 0.494 |
| Gentamicin and metronidazole as regimen backbone | 0.95 | 0.55-1.62 | 0.838 | 0.77 | 0.44-1.34 | 0.350 |
| Ciprofloxacin and metronidazole as regimen backbone (without gentamicin) | 0.94 | 0.60-1.46 | 0.785 | 0.95 | 0.60-1.49 | 0.811 |
| Incomplete regimen | 0.91 | 0.59-1.41 | 0.679 | 0.92 | 0.57-1.47 | 0.722 |
| Gentamicin dose ≤2 mg/kg  (if used) | *Reference category* | | | *Reference category* | | |
| Gentamicin dose >2 mg/kg  (if used) | 0.95 | 0.69-1.31 | 0.772 | 0.92 | 0.66-1.29 | 0.629 |

* Adjustment sex, age category, ASA class, type of surgery, duration of procedure, wound class, and transplant status. ASA; American Society of Anaesthesiologists.

**Table S5. Factors associated with odds of mortality in the 30 days after surgery**

| Variable | Univariable logistic regression | | | Multivariable logistic regression  (Adjustment for all other variables in table) | | |
| --- | --- | --- | --- | --- | --- | --- |
|  | Odds ratio | 95% confidence interval | P-value | Adjusted odds ratio | 95% confidence interval | P-value |
| Age <1 | 15.36 | 1.94-121.80 | 0.010 | 7.80 | 0.93-65.73 | 0.059 |
| Age 1-5 years | 13.04 | 1.17-145.37 | 0.037 | 15.51 | 1.26-190.11 | 0.032 |
| Age 6-25 years | 7.15 | 0.74-69.10 | 0.089 | 7.83 | 0.75-81.86 | 0.086 |
| Age 26-45 years | *Reference category* | | | *Reference category* | | |
| Age 46-65 years | 6.70 | 0.88-51.06 | 0.067 | 6.80 | 0.87-53.01 | 0.067 |
| Age 66-85 years | 15.42 | 2.12-112.12 | 0.007 | 13.81 | 1.86-102.53 | 0.010 |
| Age >85 years | 39.27 | 4.93-312.80 | 0.001 | 14.73 | 1.70-127.42 | 0.015 |
| Dirty wound | *Reference category* | | | *Reference category* | | |
| Contaminated wound | 0.39 | 0.20-0.75 | 0.005 | 0.97 | 0.46-2.02 | 0.925 |
| Clean-contaminated wound | 0.22 | 0.13-0.35 | <0.001 | 0.41 | 0.23-0.72 | 0.002 |
| Clean wound | 0.55 | 0.17-1.84 | 0.334 | 1.94 | 0.53-7.11 | 0.319 |
| ASA class 5 | *Reference category* | | | *Reference category* | | |
| ASA class 4 | 0.18 | 0.08-0.42 | <0.001 | 0.16 | 0.07-0.41 | <0.001 |
| ASA class 3 | 0.03 | 0.01-0.06 | <0.001 | 0.03 | 0.01-0.07 | <0.001 |
| ASA class 2 | 0.01 | 0.00-0.01 | <0.001 | 0.01 | 0.00-0.01 | <0.001 |
| ASA class 1 | *No events* | | | *No events* | | |

ASA; American Society of Anaesthesiologists. AUC for the multivariable model: 0.8868.

**Table S6. Factors associated with odds of acute kidney injury (AKI) in the 7 days after surgery**

| Variable | Univariable logistic regression* | | | Multivariable logistic regression*  (Adjustment for all other variables in table) | | |
| --- | --- | --- | --- | --- | --- | --- |
|  | Odds ratio | 95% confidence interval | P-value | Adjusted odds ratio | 95% confidence interval | P-value |
| Male sex | 1.42 | 1.07-1.89 | 0.016 | 1.43 | 1.06-1.92 | 0.020 |
| History of SOT or BMT | 4.19 | 2.46-7.12 | <0.001 | 2.79 | 1.52-5.11 | 0.001 |
| BMI | 1.03 | 1.01-1.06 | 0.009 | 1.04 | 1.02-1.07 | 0.001 |
| Operation duration >6 hours | 2.50 | 1.74-3.58 | <0.001 | 2.35 | 1.61-3.43 | <0.001 |
| ASA class 1 | *Reference category* | | | *Reference category* | | |
| ASA class 2 | 4.04 | 0.98-16.70 | 0.054 | 3.53 | 0.85-14.63 | 0.083 |
| ASA class 3 | 7.06 | 1.71-29.07 | 0.007 | 5.39 | 1.30-22.31 | 0.020 |
| ASA class 4 | 7.54 | 1.73-32.79 | 0.007 | 5.00 | 1.13-22.18 | 0.034 |
| ASA class 5 | 17.82 | 2.92-108.68 | 0.002 | 14.25 | 2.29-88.79 | 0.004 |
| AKI in the 7 days prior to surgery | 3.00 | 1.62-5.58 | <0.001 | 2.68 | 1.37-5.24 | 0.004 |

* Procedures undertaken prior to the introduction of Epic AKI alerts (January 2017) were excluded, as were those in which age was <25 because of an absence of AKI events. SOT; solid organ transplant. BMT; bone marrow transplant. ASA; American Society of Anaesthesiologists. AKI; Acute Kidney Injury. AUC for the multivariable model: 0.6737.

**Table S7. Factors associated with odds of newly identified MRSA infection/colonisation in the 60 days after surgery**

| Variable | Univariable logistic regression | | | Multivariable logistic regression  (Adjustment for all other variables in table) | | |
| --- | --- | --- | --- | --- | --- | --- |
|  | Odds ratio | 95% confidence interval | P-value | Adjusted odds ratio | 95% confidence interval | P-value |
| Underweight (adult BMI <18.5, or age-adjusted equivalent) | 2.06 | 0.42-9.98 | 0.370 | 1.03 | 0.21-5.10 | 0.977 |
| Normal weight (adult BMI 18.5-24.9, or age-adjusted equivalent) | *Reference category* | | | *Reference category* | | |
| Overweight (adult BMI 25-29.9, or age-adjusted equivalent) | 1.73 | 0.66-4.55 | 0.269 | 2.65 | 0.98-7.17 | 0.055 |
| Obese (adult BMI 30-39.9, or age-adjusted equivalent) | 2.22 | 0.78-6.35 | 0.137 | 3.56 | 1.19-10.62 | 0.023 |
| Severely obese (adult BMI ≥40, or age-adjusted equivalent) | *No events* | | | *No events* | | |
| In receipt of antimicrobials at CUH in the 6 months prior to surgery | 5.79 | 2.57-13.05 | <0.001 | 4.70 | 1.78-12.40 | 0.002 |
| Length of hospital stay <20 days | *Reference category* | | | *Reference category* | | |
| Length of hospital stay 20-39 days | 3.83 | 1.45-10.11 | 0.007 | 4.07 | 1.36-12.13 | 0.012 |
| Length of hospital stay 40-59 days | 7.37 | 2.29-23.77 | 0.001 | 6.59 | 1.77-24.55 | 0.005 |
| Length of hospital stay ≥60 days | 10.25 | 4.13-25.47 | <0.001 | 15.22 | 5.04-45.92 | <0.001 |
| Penicillin allergy | 2.94 | 1.30-6.64 | 0.010 | 2.82 | 1.18-6.75 | 0.020 |

Antimicrobials refers to antibacterial agents only. AUC for the multivariable model: 0.8327.

**Table S8. Factors associated with odds of newly identified VRE infection/colonisation in the 60 days after surgery**

| Variable | Univariable logistic regression | | | Multivariable logistic regression  (Adjustment for all other variables in table) | | |
| --- | --- | --- | --- | --- | --- | --- |
|  | Odds ratio | 95% confidence interval | P-value | Adjusted odds ratio | 95% confidence interval | P-value |
| Large bowel surgery | *Reference category* | | | *Reference category* | | |
| Small bowel surgery | 3.37 | 1.72-6.59 | <0.001 | 2.26 | 1.05-4.86 | 0.036 |
| Bile duct / liver/ pancreas surgery | 0.43 | 0.06-3.20 | 0.411 | 1.09 | 0.14-8.64 | 0.932 |
| Stomach / oesophagus surgery | 2.24 | 1.33-3.78 | 0.002 | 1.20 | 0.66-2.15 | 0.552 |
| Operation duration ≤120 minutes | *Reference category* | | | *Reference category* | | |
| Operation duration 121-240 minutes | 2.89 | 1.66-5.03 | <0.001 | 2.97 | 1.57-5.63 | 0.001 |
| Operation duration >240 minutes | 15.00 | 8.29-27.12 | <0.001 | 4.43 | 2.19-8.96 | <0.001 |
| In receipt of antimicrobials at CUH in the 6 months prior to surgery | 3.36 | 2.08-5.43 | <0.001 | 1.95 | 1.11-3.43 | 0.021 |
| Length of hospital stay <20 days | *Reference category* | | | *Reference category* | | |
| Length of hospital stay 20-39 days | 9.47 | 4.31-20.79 | <0.001 | 1.83 | 0.76-4.44 | 0.180 |
| Length of hospital stay 40-59 days | 22.71 | 9.66-53.40 | <0.001 | 3.50 | 1.30-9.37 | 0.013 |
| Length of hospital stay ≥60 days | 41.92 | 20.40-86.13 | <0.001 | 6.32 | 2.60-15.36 | <0.001 |
| History of SOT or BMT | 11.63 | 6.33-21.38 | <0.001 | 2.45 | 1.17-5.13 | 0.017 |
| <10 post-operative doses of antimicrobials at CUH | *Reference category* | | | *Reference category* | | |
| 11-20 post-operative doses of antimicrobials at CUH | 14.60 | 1.76-121.46 | 0.013 | 7.96 | 0.94-67.38 | 0.057 |
| 21-30 post-operative doses of antimicrobials at CUH | 54.03 | 7.12-409.93 | <0.001 | 18.73 | 2.33-150.62 | 0.006 |
| >30 post-operative doses of antimicrobials at CUH | 249.38 | 34.35-1810.45 | <0.001 | 45.44 | 5.64-365.95 | <0.001 |

Antimicrobials refers to antibacterial agents only. SOT; solid organ transplant. BMT; bone marrow transplant. AUC for the multivariable model: 0.9337.

**Table S9. Factors associated with odds of newly identified 3^rd^ generation cephalosporin-resistant Gram-negative infection/colonisation in the 60 days after surgery**

| Variable | Univariable logistic regression | | | Multivariable logistic regression  (Adjustment for all other variables in table) | | |
| --- | --- | --- | --- | --- | --- | --- |
|  | Odds ratio | 95% confidence interval | P-value | Adjusted odds ratio | 95% confidence interval | P-value |
| Operation duration > 8 hours | 5.41 | 3.28-9.11 | <0.001 | 2.53 | 1.45-4.53 | 0.001 |
| In receipt of antimicrobials at CUH in the 6 months prior to surgery | 3.59 | 2.48-5.20 | <0.001 | 1.79 | 1.19-2.69 | 0.005 |
| Length of hospital stay <20 days | *Reference category* | | | *Reference category* | | |
| Length of hospital stay 20-39 days | 7.39 | 4.57-11.95 | <0.001 | 2.77 | 1.59-4.83 | <0.001 |
| Length of hospital stay 40-59 days | 11.22 | 6.07-20.76 | <0.001 | 3.94 | 1.94-8.00 | <0.001 |
| Length of hospital stay ≥60 days | 15.78 | 9.53-26.11 | <0.001 | 4.96 | 2.68-9.20 | <0.001 |
| <10 post-operative doses of antimicrobials at CUH | *Reference category* | | | *Reference category* | | |
| 11-20 post-operative doses of antimicrobials at CUH | 6.31 | 3.02-13.20 | <0.001 | 4.19 | 1.97-8.92 | <0.001 |
| 21-30 post-operative doses of antimicrobials at CUH | 12.84 | 6.30-26.16 | <0.001 | 5.64 | 2.62-12.17 | <0.001 |
| >30 post-operative doses of antimicrobials at CUH | 27.38 | 13.81 | <0.001 | 6.77 | 3.05-15.02 | <0.001 |
| Penicillin allergy | 0.41 | 0.18-0.93 | 0.033 | 0.37 | 0.16-0.87 | 0.022 |

Antimicrobials refers to antibacterial agents only. AUC for the multivariable model: 0.8461.

**Table S10. Factors associated with odds of newly identified *Clostridioides difficile* infection/colonisation in the 60 days after surgery**

| Variable | Univariable logistic regression | | | Multivariable logistic regression  (Adjustment for all other variables in table) | | |
| --- | --- | --- | --- | --- | --- | --- |
|  | Odds ratio | 95% confidence interval | P-value | Adjusted odds ratio | 95% confidence interval | P-value |
| Length of hospital stay >60 days | 2.33 | 1.20-4.50 | 0.012 | 0.40 | 0.16-0.98 | 0.046 |
| <10 post-operative doses of antimicrobials at CUH | *Reference category* | | | *Reference category* | | |
| 11-20 post-operative doses of antimicrobials at CUH | 8.29 | 2.28-30.20 | 0.001 | 9.75 | 2.66-35.70 | 0.001 |
| 21-30 post-operative doses of antimicrobials at CUH | 5.91 | 1.41-24.82 | 0.015 | 9.46 | 2.14-41.83 | 0.003 |
| >30 post-operative doses of antimicrobials at CUH | 26.75 | 7.85-91.17 | <0.001 | 57.94 | 13.73-244.48 | <0.001 |

Antimicrobials refers to antibacterial agents only. AUC for the multivariable model: 0.8067.

**Table S11. Unadjusted and adjusted odds ratios for 30-day mortality following operations undertaken in the presence of penicillin allergy labels and with different prophylactic antimicrobial regimens**

| Variable | Univariable logistic regression | | | Multivariable logistic regression* | | |
| --- | --- | --- | --- | --- | --- | --- |
|  | Odds ratio | 95% confidence interval | P-value | Adjusted odds ratio | 95% confidence interval | P-value |
| Penicillin allergy label (any) | 0.96 | 0.47-1.92 | 0.898 | 0.90 | 0.43-1.90 | 0.782 |
| Penicillin allergy label with reaction type recorded as ‘Allergy’, ‘Unknown/unable to establish’ or left blank | 0.94 | 0.45-1.97 | 0.880 | 0.92 | 0.42-2.03 | 0.839 |
| β-lactam included in regimen | 0.70 | 0.37-1.33 | 0.279 | 0.98 | 0.49-1.95 | 0.947 |
| Broad-spectrum anaerobe cover included in regimen | 0.48 | 0.19-1.22 | 0.126 | 0.76 | 0.28-2.08 | 0.588 |
| β-lactam, metronidazole +/- gentamicin as regimen backbone | *Reference category* | | | *Reference category* | | |
| β-lactam with β-lactamase inhibitor, or carbapenem as regimen backbone | 6.34 | 2.81-14.31 | <0.001 | 3.05 | 1.29-7.25 | 0.011 |
| Gentamicin and metronidazole as regimen backbone | 3.00 | 0.62-14.55 | 0.173 | 1.95 | 0.38-10.07 | 0.427 |
| Ciprofloxacin and metronidazole as regimen backbone (without gentamicin) | 2.86 | 0.74-11.14 | 0.129 | 2.07 | 0.51-8.41 | 0.307 |
| Incomplete regimen | 8.22 | 3.03-22.26 | <0.001 | 3.03 | 1.03-8.93 | 0.045 |
| Gentamicin dose ≤2 mg/kg  (if used) | *Reference category* | | | *Reference category* | | |
| Gentamicin dose >2 mg/kg  (if used) | 0.48 | 0.11-2.16 | 0.335 | 0.29 | 0.06-1.38 | 0.120 |

* Adjustment for age category, ASA class, and wound class. ASA; American Society of Anaesthesiologists.

**Table S12. Unadjusted and adjusted odds ratios for post-operative acute kidney injury (AKI) following operations undertaken in the presence of penicillin allergy labels and with different prophylactic antimicrobial regimens**

| Variable | Univariable logistic regression | | | Multivariable logistic regression* | | |
| --- | --- | --- | --- | --- | --- | --- |
|  | Odds ratio | 95% confidence interval | P-value | Adjusted odds ratio | 95% confidence interval | P-value |
| Penicillin allergy label (any) | 0.75 | 0.47-1.22 | 0.244 | 0.73 | 0.44-1.22 | 0.232 |
| Penicillin allergy label with reaction type recorded as ‘Allergy’, ‘Unknown/unable to establish’ or left blank | 0.80 | 0.48-1.32 | 0.373 | 0.81 | 0.47-1.38 | 0.432 |
| β-lactam included in regimen | 1.35 | 0.88-2.08 | 0.169 | 1.47 | 0.92-2.34 | 0.111 |
| Broad-spectrum anaerobe cover included in regimen | 0.51 | 0.28-0.93 | 0.029 | 0.70 | 0.35-1.38 | 0.300 |
| β-lactam, metronidazole +/- gentamicin as regimen backbone | *Reference category* | | | *Reference category* | | |
| β-lactam with β-lactamase inhibitor, or carbapenem as regimen backbone | 1.64 | 1.19-2.28 | 0.003 | 1.27 | 0.89-1.81 | 0.186 |
| Gentamicin and metronidazole as regimen backbone | 0.68 | 0.30-1.50 | 0.336 | 0.65 | 0.28-1.48 | 0.302 |
| Ciprofloxacin and metronidazole as regimen backbone (without gentamicin) | 0.74 | 0.35-1.57 | 0.434 | 0.78 | 0.36-1.67 | 0.515 |
| Incomplete regimen | 2.04 | 1.17-3.55 | 0.011 | 1.15 | 0.79-2.73 | 0.223 |
| Gentamicin dose ≤2 mg/kg  (if used) | *Reference category* | | | *Reference category* | | |
| Gentamicin dose >2 mg/kg  (if used) | 1.09 | 0.69-1.71 | 0.711 | 1.18 | 0.72-1.95 | 0.507 |

* Adjustment for sex, BMI, ASA class, duration of procedure (≤6 hours or >6 hours), 7-day pre-operative AKI, and transplant status. Procedures undertaken prior to the introduction of Epic AKI alerts (January 2017) were excluded, as were those in which age was <25 because of an absence of AKI events. ASA; American Society of Anaesthesiologists. AKI; Acute Kidney Injury.

**Table S13. Unadjusted and adjusted odds ratios for newly identified VRE species infection/colonisation following operations undertaken in the presence of penicillin allergy labels and with different prophylactic antimicrobial regimens**

| Variable | Univariable logistic regression | | | Multivariable logistic regression* | | |
| --- | --- | --- | --- | --- | --- | --- |
|  | Odds ratio | 95% confidence interval | P-value | Adjusted odds ratio | 95% confidence interval | P-value |
| Penicillin allergy label (any) | 1.30 | 0.66-2.56 | 0.446 | 1.10 | 0.51-2.37 | 0.814 |
| Penicillin allergy label with reaction type recorded as ‘Allergy’, ‘Unknown/unable to establish’ or left blank | 1.31 | 0.65-2.65 | 0.457 | 1.04 | 0.47-2.33 | 0.922 |
| β-lactam included in regimen | 1.49 | 0.67-3.28 | 0.326 | 1.29 | 0.54-3.06 | 0.565 |
| Broad-spectrum anaerobe cover included in regimen | 2.90 | 0.40-21.06 | 0.292 | 5.02 | 0.62-40.51 | 0.130 |
| β-lactam, metronidazole +/- gentamicin as regimen backbone | *Reference category* | | | *Reference category* | | |
| β-lactam with β-lactamase inhibitor, or carbapenem as regimen backbone | 8.57 | 3.86-18.99 | <0.001 | 2.01 | 0.80-5.00 | 0.136 |
| Gentamicin and metronidazole as regimen backbone | 1.53 | 0.19-12.51 | 0.692 | 0.65 | 0.07-5.90 | 0.701 |
| Ciprofloxacin and metronidazole as regimen backbone (without gentamicin) | 2.91 | 0.75-11.33 | 0.124 | 2.22 | 0.51-9.65 | 0.288 |
| Incomplete regimen | 2.67 | 0.69-10.41 | 0.156 | 0.88 | 0.20-3.81 | 0.861 |
| Gentamicin dose ≤2 mg/kg  (if used) | *Reference category* | | | *Reference category* | | |
| Gentamicin dose >2 mg/kg  (if used) | 1.32 | 0.39-4.39 | 0.656 | 0.54 | 0.15-1.89 | 0.333 |

* Adjustment for type of surgery, duration of procedure, receipt of antimicrobials at CUH in the 6 months prior to surgery, number of antimicrobial doses in the 28 days after surgery, length of hospital stay, and transplant status. Antimicrobials refers to antibacterial agents only.

**Table S14. Unadjusted and adjusted odds ratios for newly identified *Clostridioides difficile* infection/colonisation following operations undertaken in the presence of penicillin allergy labels and with different prophylactic antimicrobial regimens**

| Variable | Univariable logistic regression | | | Multivariable logistic regression* | | |
| --- | --- | --- | --- | --- | --- | --- |
|  | Odds ratio | 95% confidence interval | P-value | Adjusted odds ratio | 95% confidence interval | P-value |
| Penicillin allergy label (any) | 1.27 | 0.49-3.28 | 0.621 | 1.37 | 0.52-3.59 | 0.520 |
| Penicillin allergy label with reaction type recorded as ‘Allergy’, ‘Unknown/unable to establish’ or left blank | 0.80 | 0.25-2.64 | 0.720 | 0.86 | 0.26-2.85 | 0.806 |
| β-lactam included in regimen | 1.04 | 0.40-2.71 | 0.938 | 1.01 | 0.38-2.65 | 0.989 |
| Broad-spectrum anaerobe cover included in regimen | 1.50 | 0.20-11.02 | 0.693 | 1.95 | 0.26-14.57 | 0.513 |
| β-lactam, metronidazole +/- gentamicin as regimen backbone | *Reference category* | | | *Reference category* | | |
| β-lactam with β-lactamase inhibitor, or carbapenem as regimen backbone | 3.58 | 1.51-8.49 | 0.004 | 1.62 | 0.67-3.97 | 0.287 |
| Gentamicin and metronidazole as regimen backbone | *No events* | | | *No events* | | |
| Ciprofloxacin and metronidazole as regimen backbone (without gentamicin) | 3.83 | 1.11-13.18 | 0.033 | 3.20 | 0.91-11.24 | 0.070 |
| Incomplete regimen | 0.89 | 0.11-7.22 | 0.909 | 0.47 | 0.06-3.94 | 0.490 |
| Gentamicin dose ≤2 mg/kg  (if used) | *Reference category* | | | *Reference category* | | |
| Gentamicin dose >2 mg/kg  (if used) | 2.64 | 0.66-10.60 | 0.171 | 1.68 | 0.41-6.85 | 0.472 |

* Adjustment for number of antimicrobial doses in the 28 days after surgery and length of hospital stay. Antimicrobials refers to antibacterial agents only.

**Table S15. Unadjusted and adjusted odds ratios for newly identified MRSA infection/colonisation following operations undertaken in the presence of penicillin allergy labels and with different prophylactic antimicrobial regimens**

| Variable | Univariable logistic regression | | | Multivariable logistic regression* | | |
| --- | --- | --- | --- | --- | --- | --- |
|  | Odds ratio | 95% confidence interval | P-value | Adjusted odds ratio | 95% confidence interval | P-value |
| Penicillin allergy label (any) | 2.97 | 1.31-6.72 | 0.009 | 2.82 | 1.18-6.75 | 0.020 |
| Penicillin allergy label with reaction type recorded as ‘Allergy’, ‘Unknown/unable to establish’ or left blank | 2.83 | 1.21-6.65 | 0.017 | 2.60 | 1.05-6.45 | 0.039 |
| β-lactam included in regimen | 0.42 | 0.18-0.97 | 0.042 | 0.50 | 0.20-1.27 | 0.146 |
| Broad-spectrum anaerobe cover included in regimen | 1.19 | 0.16-8.86 | 0.863 | 1.35 | 0.17-10.52 | 0.777 |
| β-lactam, metronidazole +/- gentamicin as regimen backbone | *Reference category* | | | *Reference category* | | |
| β-lactam with β-lactamase inhibitor, or carbapenem as regimen backbone | 3.13 | 1.12-8.83 | 0.030 | 1.81 | 0.55-6.00 | 0.332 |
| Gentamicin and metronidazole as regimen backbone | 4.42 | 0.85-22.01 | 0.077 | 3.69 | 0.63-21.46 | 0.146 |
| Ciprofloxacin and metronidazole as regimen backbone (without gentamicin) | 4.08 | 0.97-17.19 | 0.055 | 3.17 | 0.67-15.01 | 0.146 |
| Incomplete regimen | 3.70 | 0.88-15.59 | 0.074 | 1.83 | 0.32-10.58 | 0.498 |
| Gentamicin dose ≤2 mg/kg  (if used) | *Reference category* | | | *Reference category* | | |
| Gentamicin dose >2 mg/kg  (if used) | 0.87 | 0.18-4.34 | 0.869 | 0.85 | 0.16-4.55 | 0.847 |

* Adjustment for BMI category, type of surgery, receipt of antimicrobials at CUH in the 6 months prior to surgery, and length of hospital stay. Antimicrobials refers to antibacterial agents only. CUH; Cambridge Univesity Hospitals NHS Foundation Trust.

**Table S16. Unadjusted and adjusted odds ratios for newly identified 3^rd^ generation cephalosporin-resistant Gram-negative infection/colonisation following operations undertaken in the presence of penicillin allergy labels and with different prophylactic antimicrobial regimens**

| Variable | Univariable logistic regression | | | Multivariable logistic regression* | | |
| --- | --- | --- | --- | --- | --- | --- |
|  | Odds ratio | 95% confidence interval | P-value | Adjusted odds ratio | 95% confidence interval | P-value |
| Penicillin allergy label (any) | 0.41 | 0.18-0.93 | 0.033 | 0.37 | 0.16-0.87 | 0.022 |
| Penicillin allergy label with reaction type recorded as ‘Allergy’, ‘Unknown/unable to establish’ or left blank | 0.38 | 0.15-0.94 | 0.036 | 0.34 | 0.13-0.86 | 0.022 |
| β-lactam included in regimen | 1.10 | 0.63-1.92 | 0.740 | 1.15 | 0.64-2.06 | 0.636 |
| Broad-spectrum anaerobe cover included in regimen | 0.46 | 0.23-0.94 | 0.032 | 0.67 | 0.32-1.42 | 0.297 |
| β-lactam, metronidazole +/- gentamicin as regimen backbone | *Reference category* | | | *Reference category* | | |
| β-lactam with β-lactamase inhibitor, or carbapenem as regimen backbone | 2.61 | 1.65-4.12 | <0.001 | 1.10 | 0.67-1.81 | 0.693 |
| Gentamicin and metronidazole as regimen backbone | 0.75 | 0.18-3.20 | 0.702 | 0.45 | 0.10-1.99 | 0.292 |
| Ciprofloxacin and metronidazole as regimen backbone (without gentamicin) | 0.24 | 0.03-1.74 | 0.156 | 0.19 | 0.02-1.40 | 0.102 |
| Incomplete regimen | 3.01 | 1.54-5.89 | 0.001 | 1.29 | 0.63-2.65 | 0.485 |
| Gentamicin dose ≤2 mg/kg  (if used) | *Reference category* | | | *Reference category* | | |
| Gentamicin dose >2 mg/kg  (if used) | 1.63 | 0.83-3.19 | 0.156 | 0.82 | 0.40-1.65 | 0.574 |

* Adjustment for duration of procedure, receipt of antimicrobials at CUH in the 6 months prior to surgery, number of antimicrobial doses in the 28 days after surgery, and length of hospital stay. Antimicrobials refers to antibacterial agents only. CUH; Cambridge University Hospitals NHS Foundation Trust.
